# The Managerial Implications of the Key Performance Indicators in Healthcare Sector: A Cluster Analysis

**DOI:** 10.3390/healthcare9010019

**Published:** 2020-12-25

**Authors:** Adriana Burlea-Schiopoiu, Koudoua Ferhati

**Affiliations:** 1 Management, Marketing, Business Administration, Faculty of Economics and Business Adminsitration, University of Craiova, Strada Alexandru Ioan Cuza 13, 200585 Craiova, Romania; 2 AVMF Research Laboratory, University of Salah Boubnider, Constantine 3, Constantine 25000, Algeria; koudoua.ferhati@univ-constantine3.dz

**Keywords:** key performance indicators, healthcare system, pandemic crisis, COVID-19, Algeria

## Abstract

The aim of the paper is to identify a set of the key performance indicators (KPIs) in order to provide managers and employees from the healthcare system with recommendations for evaluating, monitoring, and controlling the critical factors that influence the performance of the healthcare sector in Algeria during a pandemic crisis. During February–August 2020, a cross-sectional survey design was administrated to medical employees from hospitals situated in the northeastern part of Algeria. Our findings proved that the four groups of KPIs correlate to each other, and during this period, the triple relationship among human factor-technology-medication plays a decisive role in reducing the pressure on the medical system and overcoming the crisis. In order to increase the efficiency of the decision-making process, a hierarchy of KPIs is recommended in terms of their impact on the performance of medical staff. The practical importance of our research consists in ranking KPIs on four clusters that support managers to focus on both the human factor (clinical errors, infection rate, and medication errors) and the technical elements of maximum importance (laboratory test time, location of the facility, and sufficient air).

## 1. Introduction

The recent crisis that the entire world is facing caused by COVID-19, a pandemic that has forced all organizations, whether public or private, to rethink their mission and vision. Thus, the efficacy of the healthcare sector depends greatly on the rapidity to adapt to the new dramatic situation. Before the World Health Organisation (WHO) had declared the COVID-19, a pandemic crisis, on 11 March 2020, Algeria had the situation under control, although the first two cases were registered on 25 February 2020. The rapid evolution of the pandemic coronavirus crisis requires that the common strategies should be oriented toward ensuring the health of the population and a continuous assessment of the events to give priority to future needs. The situation in Algeria is not far from the rest of the world, with a total of 55,081 cases and 1880 deaths recorded until 22 October 2020, with a mortality rate of 11.70% [1]. This high rate represents a threat to the national health situation of the country that leads to making a study to understand how the healthcare sector is dealing with this crisis and what are the available and efficient managerial tools to help managers and healthcare staff to better control facilities [2].

The international statistics of WHO [3] place Algeria in the fourth position in Africa (with 50,914 cases) after South Africa (with 669,498 cases), Ethiopia (with 72,700 cases), and Uganda (with 7364 cases) with a total of 1,172,342 confirmed cases and 25,481 deaths in the continent as the last update.

Watkins et al. [4] analysed the impact of the pandemic situation on SMEs from Australia and they observed that only six per cent of Australian SMEs had a plan for avoiding the pandemic crisis. Thus, the other 39 per cent consider that the pandemic has no impact on their business and over 60 percent called upon the need to prepare a viable strategy to tackle a pandemic situation. As a result, in 2007, the Australian SMEs were not prepared for a pandemic situation and 13 years later SMEs worldwide are in the same situation.

Watkins et al. [5] considered that a strategy for the pandemic situation among SMEs depends on the perception of the risk level and the resources that are available to prepare and frame the strategy.

Ivo Hristov and Antonio Chirico [6] identified the impact of key performance indicators (KPIs) on company performance in the framework of sustainable strategies and they arrived at the conclusion that existing literature does not provide ample evidence about how to address the crises from managerial perspective.

At this moment, healthcare managers, officials, and policymakers are seeking to answer the following questions:

How will we manage to streamline the healthcare sector in real-time to overcome this pandemic generated by the novel coronavirus?

Can KPIs be used as a tool enabling a quick reaction of the healthcare sector’s facilities to the adverse effects of COVID-19?

What is the possible set of KPIs that leaders in the healthcare sector are required to check in order to deal with the consequences of the virus and re-establish “normality” in the social and business environment?

The answers to these questions were found in the last months from many scholars around the world from economic point of view, but not from the healthcare sector perspective [7,8,9,10]

These analyses motivated us to approach this pandemic crisis from another perspective based on KPIs as instruments of human competencies in the fight against this enemy.

The range of factors affecting sustainability performance is varied, from the small details to the big issues in healthcare facilities. Thus, most of them are not obvious to the decision makers (e.g., maintenance and building design) because of lack of technical skills (e.g., attention towards the aspects of bioclimatic passive strategies and incorrect architectural and flexible layouts, which often limit the clinical processes and causes the decrease of performance and sustainability criteria) [11].

The use of KPIs as tools to improve the effect of public health measures and as indicators of the measures that need to change in function of true morbidity and mortality rates.

## 2. Literature Review and Premises of Research

Mohamed Khalifa and Parwaiz Khalid [12] classified KPIs on three levels of performance (operational, tactical, and strategic indicators), six levels of performance dimensions (safety, effectiveness, efficiency, timeliness, patient-centeredness, and equity) and three levels of system components (structure, processes, and outcomes). They have identified fifty-eight KPIs, classified into ten categories.

According to the literature review and based on our preliminary research, considering the particularities of the pandemic COVID-19, 41 indicators were selected for the KPI of the healthcare system in Algeria. The structure of the KPI is the following: ten indicators for social sustainability, eight indicators for economic sustainability, nine indicators for the internal process and fourteen indicators for the technical domain.

### 2.1. Social Sustainability Indicators

Social sustainability indicators (SSI) for healthcare facilities facing a crisis can be ambiguous to define and apply. Vallance et al. [13] (p. 342) affirm that SSI is “a concept in chaos”. SSI have been organized under the broad categorical concerns of well-being, values, agency, and inequality [14].

Some researchers consider that SSI can be used to measure the performance in a state of overall well-being [15], and that KPIs should mainly contribute to improvement of the people’s life [16].

In consequence, we need to be very specific while setting the indicators for social sustainability that helps track the critical metrics within a facility during a global pandemic crisis. As a result, there have been many attempts to categorize the various approaches to social sustainability indicators (Appendix A).

### 2.2. Economic Sustainability Indicators

The assessment of sustainability performance is assumed to be appropriate to the healthcare industry. Many studies were carried out with the scope of identifying an initial set of potential KPIs from an economic perspective [17] that could be used for sustainability performance evaluation to keep facilities operating with the minimum regular conditions of sustainability while facing the global pandemic (Appendix B).

A detailed explanation of ESI from a company point of view (Novo Nordisk A/S) was realized by Morsing et al. [18] and they arrived at the conclusion that KPIs are related to social and economic objectives, but are reflected on internal environment and also external environment.

### 2.3. Indicators of Internal Process

The indicators of internal process (IIP) in healthcare facilities are a well-defined performance measure used to monitor, analyse, and optimize all relevant processes and practices of the facility’s staff to increase patient satisfaction and diminish any possible errors or damages (Appendix C).

### 2.4. Technical Indicators

Because of the complex technical and architectural nature of healthcare facilities, it requires a special set of indicators that suit the specific strategic objective’s action plan. When facing a global pandemic such as COVID-19 every detail becomes important and overlooking, even the thinnest element such as insulation or air quality may lead to losing control over the crisis (Appendix D).

The KPI’s constitute a management control tool that is used for planning and prioritizing actions, for making decisions and responding to problems in real time [19,20]. Elmar Hörner [21] arrived at the conclusion that one of the most important KPIs for measuring the success of the areas that need improvements in the pharmaceutical domain (e.g., Merck KGaA) is “Decision Making”. The continuous monitoring of KPIs makes possible to answer the question of whether the objectives are achievable and, if yes, to what extent, which constitutes the basis for evaluation of the performance of contemporary facilities.

As the main role of a KPIs’ system is tracking the performance and sustainability in the healthcare facility for the interest’s spot; thus, it is fundamental to make an analysis of the facility’s needs, strategy and goals. Therefore, in the case where the urgent need of crisis management is the main strategy, KPIs should serve this objective.

According to Schmidt et al. [22] (p. 760): “In order to successfully create new KPIs, it is crucial to analyse and understand the underlying cause and effect relationships as well as interdependencies between processes, equipment, and energy efficiency”.

Recently, Daria Mikhailova [23] has chosen the KPIs to assess the performance of Pharmaceutical Project Management Quality System Effectiveness and she arrived at the conclusion that KPIs proved a practical support for both system monitoring and system interventions. Moreover, David Parmenter [20] underlines the importance of KPIs selection in the healthcare system and he advised us never underestimate the negative consequences of the choice of an inadequate KPIs.

Fernandes et al. [24] stated that all panellists finally accepted only eight KPIs. We allocated the eight KPIs of Fernandes et al. to our KPIs classes (e.g., SSI: providing in-person disease and medication education to patients; participating in interprofessional patient care rounds; providing discharge patient medication education; ESI: performing discharge medication reconciliation; and IIE: performing admission medication reconciliation (including best-possible medication history); completing pharmaceutical care plans; resolving drug therapy problems; and providing bundled, proactive direct patient care activities).

Matsuoka and Hirai [25] used the KPIs for explaining the core principles of Society 5.0 based on three factors (structural transformation, technological innovation, and quality of life). In addition, Re Cecconi et al. [26] used the KPIs to underline the importance of the technical indicators as strategic tools for improving the decision-making process. Jiang et al. [27] starting from the premise that performance measurement is of vital importance for the healthcare systems, especially during crisis periods, they proposed a model based on KPIs to help managers to make good decisions under time pressure. In the same line, Niemi et al. [28] consider the mean lead-time (MLT) as one of the most important KPI.

Ramzi Shawahna [29], (p. 2) affirms that KPIs “are often developed for capturing the performance of healthcare providers and the provision of services. These KPIs are supposed to monitor if healthcare services were provided with consistency and efficiency”.

Based on both theoretical and practical arguments, we propose the following hypotheses:

**Hypothesis** **1.** *The economic sustainability indicators directly influence the indicators of internal process*.

**Hypothesis** **2.** *The social sustainability indicators directly influence the economic sustainability indicators.*

**Hypothesis** **3.** *The social sustainability indicators directly influence the indicators of internal process*.

**Hypothesis** **4.** *The technical indicators directly influence the economic sustainability indicators*.

**Hypothesis** **5.** *The technical indicators directly influence the indicators of internal process*.

**Hypothesis** **6.** *The technical indicators directly influence the social sustainability indicators.*

## 3. Methodology

To select the KPIs, we have conducted in-depth interviews with four health management experts and one assistant professor from the Faculty of Medicine of Constantine 3 Salah Boubnider University to discuss and identify a list from the extracted indicators of literature review (185). The first set of indicators was developed from the literature review: 158 indicators were extracted from the discussions with the experts and were selected, then 62 indicators were divided into four main domains for sustainability assessment: social, economic, technical, and internal processes. The selected indicators were used to design the questionnaire for the first round of the Delphi method.

In the second stage, in consensus with Galanis [30], we employed the Delphi method in two rounds. In the first round, 20 public hospitals and managers for each hospital have been chosen to participate in the research, as they were involved in sustainability. The questionnaires were distributed to managers to investigate the implication of KPI’s for primary healthcare facilities’ sustainability performance.

In the second round, we distributed the questionnaires to the same hospitals’ managers, and, in this stage, we eliminated the indicators with the lowest mean scores. After we received the feedback from the managers, we reduced the number of KPIs (we eliminated 22 indicators) and 41 KPIs were included in the final setup of our research.

This quantitative study has been conducted from the first week of recording the first three cases of COVID-19 disease in Algeria until the end of August 2020; investigation was made in two main wilayas: El Taref and Constantine, including different type of facilities: university hospital, public hospital centers, and neighbourhood’s healthcare facilities.

During February–August 2020, a total of 300 questionnaires were distributed to Algerian hospitals that were identified as very important hospitals in the framework of the pandemic coronavirus. In total, 210 completed responses (response rate of 70 percent) were collected from medical staff who are directly involved in the battle with this invisible enemy. Respondents were required to evaluate the importance of every KPIs using a five-point Likert scale (1—not important at all to 5—very important). 

We chose to use the SmartPLS [31] method to analyse the data and we started the research by assessing the measurement model to ensure that each construct’s KPIs are reliable and valid.

### 3.1. Sample

The 88 respondents (41.9%) were from facilities situated in El Taref and 122 respondents (58.1%) from facilities situated in Constantine. The gender of the respondents was balanced with 96 females (45.7%) and 114 males (54.3%). The age of the respondents was distributed as follows: 21.4% between ages of 25 and 35 years, 37.6% between ages of 36 and 45 years, 24.3 between ages of 46 and 55 years, and 16.7% between ages of 56 years and over (M = 2.36; SD = 0.999).

A total of 109 respondents participated in the pilot study and at the final of this study all 41 KPIs were retained because they registered a loading factor above the threshold of 0.70 (Appendix E), in consensus with Sarstedt et al. [32].

### 3.2. Measures

We started the research by assessing the measurement model to ensure that each construct’s KPIs are reliable and valid.

Internal consistency of the research model was assessed by partial least squares structural equation modelling (PLS-SEM), we started by examining the indicator loadings [32], ranging from 0.701 to 0.866 and indicating that the KPIs have a very good degree of reliability (Appendix E).

Next, we calculated the “reliability indicators” and higher values indicate increased levels of reliability. The main indicators exceed the minimum threshold of 0.7 [33] as follows: Cronbach’s Alpha that measures internal consistency reliability ranged from 0.903 to 0.932 and represent good to very good reliability levels of KPIs, Dijkstra-Henseler’s rho_A ranged from 0.907 to 0.934 [34] and composite reliability (CR) ranged from 0.920 to 0.940. Thus, all the values exceed the minimum threshold value of 0.7 for all variables indicating that the measurement model has good reliability (Table 1).

The descriptive statistics indicate the values of the mean and we observe that a mean value of 4.01 out of 5 suggests that most of the respondents mainly agreed that IIP is very important KPIs for the healthcare system in a pandemic situation. Meanwhile, the SSI registered the lowest value (3.86) and an explanation consists of the particularity of this period and on pressure existing on medical personnel.

The pressure is reflected on value of loading factor of the IIP-C6 (Medical errors = 0.860), followed by IIP-C5 (Clinical errors = 0.847); IIP-C1 (Medication errors −0.822); IIP-C3 (Mortality rate = 0.789); IIP-C4 (Infection rate = 0.770) and finally IIP-C9 (Laboratory test time = 0.720).

The standard deviation shows that there are no relevant differences among the KPIs as the values are close to one another for ESI, IIP, and TI (ranged from 0.648 to 0.673); only SSI registered a value above 0.770.

We evaluated the extent to which any selected construct differs from the others and we tested the “convergent validity” and we used the average variance extracted values that are greater than 0.5 (from 0.530 to 0.622) and validate the latent variables for our model composition, in consensus with Hair et al. [35].

Average variance extracted (AVE) analysis was conducted for evaluating if we have a good convergent discriminant validity and if each construct exceeds the threshold value of 0.50. The result proves that all KPIs are retained.

The results prove that the indicators of internal process have the highest value of Cronbach alpha (0.918) which highlights the importance of this group of indicators in the context of the current pandemic coronavirus crisis.

We continued to assess the “discriminant validity” by calculating the Fornell and Larcker [36] criterion and for proving the relevance of the structural model. The highest correlation was registered between ESI-ESI (0.788) and the lowest correlation was registered between ESI-SSI (0.423).

In order to consolidate the assessment of the discriminant validity in variance-based structural equation modelling we used Heterotrait–Monotrait ratio (HTMT), which is considered superior to previous indicators [37] as Fornell–Larcker criterion and (partial) cross-loadings (Table 2).

The results show that the values of HTMT were smaller than 0.90 (ranged from 0.420 to 0.848), which means that this ratio meets the requirements of the Henseler et al. [38]. In order to sum, the model assessments prove a good evidence of validity and reliability.

## 4. Results and Discussion

The correlation between KPIs was used for verifying the relationship between all variables (Table 3). 

The correlations between variables reveal to us that the age of respondents negatively influences three KPIs (IIP, ESI, and TI) and prove that under pressure the experience of the medical personnel is important for the decision-making process. The age of respondents is positively correlated with patient satisfaction, because no matter their age, the healthcare system employees are devoted to their job and to their patients.

The gender of respondents and the location directly and negatively influence two KPI’s (ESI and TI) and directly and positively influence the other two KPIs (SSI and IIP). These correlations prove that the practical KPIS as ESI and TI are perceived as having a negative influence on decision-making process under pressure.

The location is an important variable, because the patients with COVID-19 are in some regions and they are treated in hospitals specially designated for this disease. Algeria has a public healthcare sector and it is accessible and free of charge for all citizens, financed by the government, given Algeria’s young population. In close alignment with this long-term strategy, the government maintains an intensive immunization program.

The correlations between IIP and the other three KPIs are positive and prove that internal process is developed for the purpose of patient satisfaction (0.783) and considering the consequences of the infection with COVID-19, IIP is related to facilities of the hospital (0.685) in terms of qualitative and especially, quantitative KPIs.

We analyse the results and we first test the collinearity of the research model and we observe that the variance inflation factor VIF values ranged from 1.897 (Hospital readmission rate) to 3.852 (Indoor air quality) and is within the limits recommended by Hair et al. [35].

**Figure 1:**
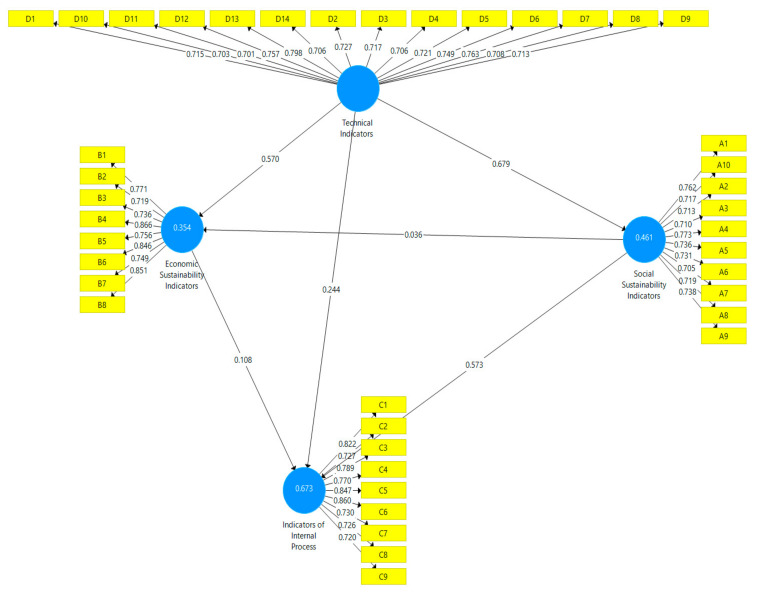
The Path Coefficients of the research model for KPIs. Source: Authors’ contribution based on SmartPLS.

The results prove that there is no collinearity problem interfering with our KPIs and we continued to evaluate the research model by interpreting the coefficient of determination (R2), *f*^2^, and *P*. The coefficient of determination between 0.25 and 0.50 is considered good and above 0.50 are considered very well. Figure 1 shows values of R^2^, ranged from 0.254 to 0.673. In conclusion, the predictive power of the model and R^2^.

We arrived at the conclusion that all the KPIs are valid and reliable and we assess the research model and test the hypotheses (Table 4).

The resulting effect size value of each KPI in the model ranges from 0.001 to 0.855, which are included in the category of very small to large [39]. The value of goodness of fit that is generated through the standardized root mean squared residual (SRMR) is equal to 0.08, which means that our model fits the empirical data [40]. We also tested our hypotheses with the coefficient parameter and the significant value generated from the 95% bias-corrected confidence intervals of each KPI.

The path coefficients provide significant value (at the *p* 0.05 level), only the relationship SSI -> ESI is not supported. Thus, the value of the coefficient (T) to the relationship SSI -> ESI is 0.491 with a *p*-value < 0.623. In conclusion, all hypotheses are supported except for the second hypothesis.

The particularities of our study due to the pandemic crisis do not allow us to affirm that our results support previous studies, because not many studies related to KPIs were developed during the pandemic crisis.

We can link these results to the pandemic crisis when the communication process and human factors are more important than economic and material factors. Hospital performance is a reference to key performance indicators (KPIs), especially to IIP and TI as promoters of ESI and SSI, because in this period quantitative assessments of hospitals became an indicator of the capacity of them to achieve the new goals by making efficient use of the limited resources available in the crisis period. This signifies that TI can be used to improve SSI and the ESI have a positive effect on IIP.

Healthcare facilities all over the world are dealing with major challenges to keep operating in a performing way during crisis time especially when it’s facing a world pandemic such as Covid-19. This pandemic coronavirus took by surprise both the decision-makers and the employees who faced an unpredictable enemy and impossible to be controlled. The fact that many organizations, public or private, were forced to discontinue their activity for an unspecified period of time has created a state of panic and uncertainty, upsetting society at all levels.

Our findings proved that KPIs play an important role in increasing the performance of healthcare systems, and, especially during the pandemic coronavirus crisis (Appendix F).

**Figure 2:**
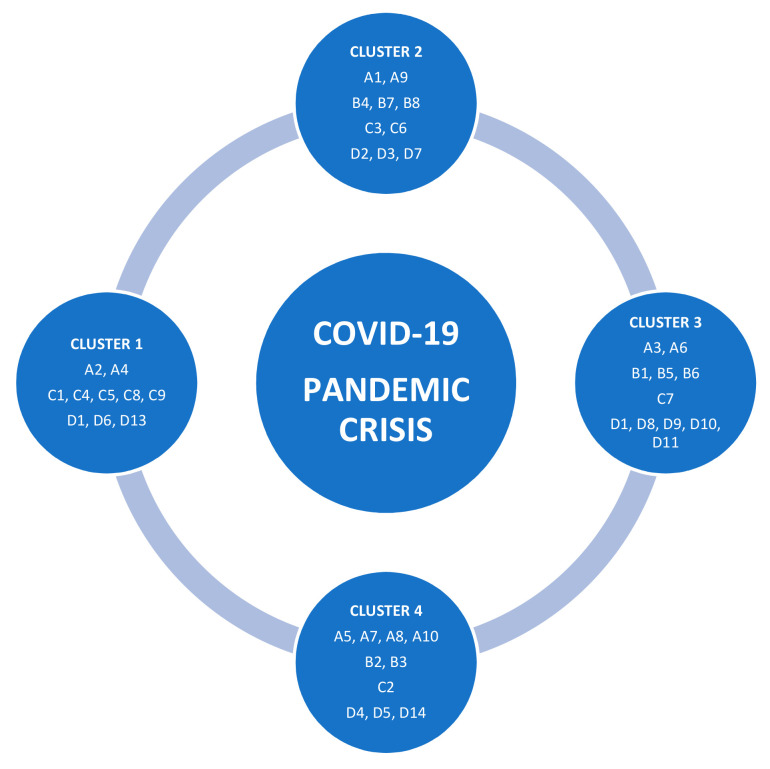
The Clusters of the key performance indicators (KPIs) as a managerial decision support during pandemic crisis. Source: Authors’ own contribution

Figure 2 shows the four clusters of KPIs that can be a priority for hospital managers in a crisis period and every cluster includes different KPIs ranked by importance.

The first cluster includes 10 KPIs, two KPIs of SSI (related to the average hospital stay and on patient waiting time), five KPIs of IIP (related to clinical and medication errors, infection rate, average length of stay in the emergency room, and laboratory test time), and the other three KPIs are from TI as indoor air quality, sufficient air conditioning, and location of the facility.

This cluster proves the importance of this indicator in a crisis because under pressure and in very stressful conditions the decisions made by doctors concerning medical prescriptions or dozes might be mistaken [41]. Clinical errors tracking and assessment appears to be significant in crisis time and can affect the medical staff and equipment’s effectiveness. The sufficient air conditioning allows checking the sufficiency of air out of the facility’s HVAC system to ensure the well-being of staff and patients inside the healthcare facility. This indicator is crucial for the patients because one of the causes of death is insufficiency respiratory. Waiting time in the emergency room shows the value of assessing this metric in order to set less waiting time targets in crisis time.

These findings are in consensus with the findings of Mohamed Khalifa and Parwaiz Khalid [12] who considered that patient safety and infection control rates are very important indicators to gauge the quality of the healthcare system.

The second cluster comprises the other 10 KPIs as follow: two KPIs of SSI that measure the patient satisfaction and patient transfer rate to other facilities, three KPIs of ESI that indicate average care costs of insured patients, cost of work-staff and current cost per bed; two KPIs of IIP related to mortality rate and to medical errors, and three KPIs of TI concerning natural light penetration, vertical circulation and degree of thermal comfort. Patient satisfaction measures the degrees to which the medical service responds to patients’ expectations is a high priority for the management strategy in a pandemic crisis. Mortality rate measures the rate of deaths while in a world pandemic and it is considered a structural tool for decision-making and setting the facility’s strategy to deal with the crisis according to the given number. The degree of thermal comfort could affect or be affected by other technical and internal processes indicators related to the wellbeing inside the facility.

The third cluster includes 11 KPIs structured by categories as following: two KPIs of SSI (related to hospital readmission rate and to waiting time in the emergency room); three KPIs of ESI including average hospital expenses, cost of drugs, and equipment and also average care costs; one KPI of IIP (waiting time for admission to the operating room), and five KPIs of TI (waste management, energy and emissions control, quality of the building envelope, artificial lighting, and water consumption).

The cost of drugs and equipment needs to be taken into consideration because of the increasing of needed medicines, supplies, and special equipment as in a crisis, and to be correlated to the patient waiting time in a crisis that is also important because every patient needs medical care as soon as possible when he/she arrives at the facility to avoid any complications could expose his/her lives to danger. Quality of the building envelope plays a key role in the healthcare environment inside the facility as it controls directly other technical indicators ranked.

The fourth cluster includes 10 KPIs as follows: four KPIs of SSI (patient safety, rate of vacant patients in beds, patient complaint rate, and the number of new patients); two KPIs of ESI concerning average maintenance costs and costs per payer; one KPI of IIP related to bed occupancy rate and three KPIs of TI (acoustic insulation, distributions of medical devices, and hierarchy of functional spaces). The rate of vacant patients in beds allows the facility managers to set their priorities and to make decisions like patients transfer to other departments or facilities. The number of new patients indicates the particularity of this pandemic crisis because it is limited to receive more patients in the facility, because of the virus, which needs to be followed to make sure that the facility is ready for offering care to an expected number of patients. The hierarchy of functional spaces underlines every daily movement of the staff between rooms, controlling, services, and departments. As a result, such a metric gives more flexibility and performance in hard times where every second counts inside the healthcare facility.

The respondents considered as very important two indicators: infection rate and patient safety, because controlling infection rates and applying protocols in healthcare facilities is considered a key practice when facing pandemics. In this situation, patient complaint rate, like the patient’s satisfaction, is not a high priority for healthcare facilities in a pandemic situation.

Waiting time for admission to the operating room gives the facility’s managers a clear vision about the target time to set surgical operations to increase the internal processes performance, going to cost of work-staff that is considered as a lower priority in these circumstances.

Laboratory test time was not considered as a high priority indicator, and this might be justified by the healthcare facilities protocols in case of a pandemic crisis as test samples and results are oriented to the big test labs in the country such as Pasteur Institute.

Our results confirm the findings of McCance et al. [42] that analysed the eight KPIs clustered within the person-centered processes domain of the framework, and these KPIs were related to patient satisfaction, confidence, and implication in the decision-making process about his/her care.

To prepare action plans for in time strategies and to implement them successfully, the healthcare facilities continue to look for appropriate strategy implementation tools. Consequently, the measurement systems are used to evaluate the effects of the healthcare facilities actions. The role of such system is to support the management process as well as the process of implementation of the hospital’s strategy, which should include not only solid technical and economic factors (particularly financial measures), but also the requirements of the corporate social responsibility and sustainable development standards, as well as the employee relations and value management requirements [43].

One solution is to use KPIs and keep controlling performance and sustainability through dashboards and scorecards to make frequent and continuous evaluation for the outputs to ensure a better crisis management for healthcare facilities and to make the strategic objectives to be achieved clearer and more convenient. In the case of Algeria, we did not find previous studies about KPIs for evaluation in healthcare facilities from a managerial perspective. For this reason, we consider this study important and it can add a real value to the research, because it helps the managers to evaluate sustainability performance of healthcare facilities in crises.

## 5. Conclusions

Our findings prove the opportunity for healthcare system employees and not only for hospital managers, to identify critical KPIs in a short period of time and with lower costs. It is very important to consider that the IIP are situated in the first place, which gives us an idea about the priorities of healthcare staff in a crisis. We recommend focalizing the improvements in the areas with high potential to propagate the factors of the pandemic crisis.

In the framework of a pandemic crisis, the performance of healthcare systems is related to its capacity to quickly respond to danger generated by COVID-19. In this period, it is difficult to adapt to an existing model, because the variables are completely different, and the weaknesses of hospitals are also different.

The practical implications are underlined by our model that provides hospitals’ manager’s solutions for the decision making process under pressure indicating the ways of improvements of quality of medical services by implementing suitable KPIs. Thus, these clusters of KPIs can be used as tools for developing sustainable healthcare systems not only in Algeria but also in developing countries that need financial material and human support to overcome the pandemic crises.

Our study fills the gap in the literature concerning the correlations between KPIs in the healthcare sector during a pandemic crisis. Moreover, the managers can establish realistic goals by using KPIs taking into account their level of importance as early-warning indicators that can point out forthcoming changes in the evolution of the crisis. Healthcare managers can use the clusters KPIs to evaluate executive performance and to develop strategies for saving lives.

In the last years, the KPIs were found as representative of overall healthcare systems around the world [24]. Paradoxically, the qualitative differences between healthcare systems around the world are reduced by the particularities of the crisis, because a crisis is a negative phenomenon at globally level and its repercussions are more or less evaluated by KPIs [27]. Moreover, during a pandemic crisis, the information plays an important role in reducing physiological and mental impact on the people [44] and for this reason, it is necessary to use the KPIs clusters in a flexible way and to adapt them to healthcare facilities [45].

The results could provide a guide to hospitals’ decision-makers in order to have under control the situation of the Algerian healthcare system [46] and for the other countries healthcare systems, because our findings are in consensus with the results of the other researchers [47,48,49]

The limitations of our study are related to the sample size, because, considering the short period of analysis, we used a relatively small sample size, from Algerian hospitals and the findings should be critically analysed by considering the specificity of Algeria.

This study only analysed the relationship between four groups of KPIs without testing the direct impact of these KPIs on the performance of the Algerian hospitals.

Our Clusters of KPIs model can be adapted to healthcare systems from different countries, but it is important that every healthcare facility choose its own KPIs taking into account their human, financial and technical resources [50].

Future research should be oriented to testing the role of KPIs in the improvement of the hospitals’ performance in relationship with the social responsibility and with the improvements of the commitment of the healthcare systems employees.

## Figures and Tables

**Table 1:** Descriptive Statistics, and Reliability and Validity of Measurement Model.

KPIs	Mean	Std. Deviation	Cronbach’s Alpha	rho_A	Composite Reliability (CR)	Average Variance Extracted (AVE)
**ESI**	3.86	0.648	0.914	0.933	0.929	0.622
**IIP**	4.01	0.670	0.918	0.922	0.932	0.606
**SSI**	3.81	0.776	0.903	0.907	0.920	0.534
**TI**	3.83	0.673	0.932	0.934	0.940	0.530

Source: Authors’ own contribution based on SmartPLS.

**Table 2:** Heterotrait–Monotrait Ratio (HTMT) Test.

KPIs	Heterotrait–Monotrait Ratio (HTMT)				Fornell–Larcker Criterion			
	ESI	IIP	SSI	TI	ESI	IIP	SSI	TI
**ESI**	-	-	-	-	0.788	-	-	-
**IIP**	0.511	-	-	-	0.496	0.778	-	-
**SSI**	0.420	0.848	-	-	0.423	0.784	0.731	-
**TI**	0.608	0.739	0.714	-	0.594	0.697	0.679	0.728

Source: Authors’ own contribution based on SmartPLS.

**Table 3:** Correlations between KPIs.

Variables	Correlations	Age	Gender	Location	SSI	ESI	IIP	TI
Age	Pearson Correlation	1	0.050	0.192 **	0.084	−0.069	−0.005	−0.059
	Sig. (2-tailed)		0.467	0.005	0.226	0.320	0.948	0.392
	*N*	210	210	210	210	210	210	210
Gender	Pearson Correlation	0.050	1	−0.189 **	0.092	−0.065	0.001	−0.028
	Sig. (2-tailed)	0.467		0.006	0.185	0.348	0.986	0.689
	*N*	210	210	210	210	210	210	210
Location	Pearson Correlation	0.192 **	−0.189 **	1	0.060	−0.101	0.063	−0.060
	Sig. (2-tailed)	0.005	0.006		0.386	0.146	0.364	0.388
	*N*	210	210	210	210	210	210	210
SSI	Pearson Correlation	0.084	0.092	0.060	1	0.392 **	0.783 **	0.666 **
	Sig. (2-tailed)	0.226	0.185	0.386		0.000	0.000	0.000
	*N*	210	210	210	210	210	210	210
ESI	Pearson Correlation	−0.069	−0.065	−0.101	0.392 **	1	0.475 **	0.562 **
	Sig. (2-tailed)	0.320	0.348	0.146	0.000		0.000	0.000
	*N*	210	210	210	210	210	210	210
IIP	Pearson Correlation	−0.005	0.001	0.063	0.783 **	0.475 **	1	0.685 **
	Sig. (2-tailed)	0.948	0.986	0.364	0.000	0.000		0.000
	*N*	210	210	210	210	210	210	210
TI	Pearson Correlation	−0.059	−0.028	−0.060	0.666 **	0.562 **	0.685 **	1
	Sig. (2-tailed)	0.392	0.689	0.388	0.000	0.000	0.000	
	*N*	210	210	210	210	210	210	210

**. Correlation is significant at the 0.01 level (2-tailed); Source: Authors’ own contribution based on SmartPLS.

**Table 4:** Path Coefficients.

Relations	Original Sample (O)	Sample Mean (M)	Standard Deviation (STDEV)	T Statistics (|O/STDEV|)	*f* ^2^	*p*-Values	Confidence Intervals		Conclusion
							2.5%	97.5%	
**ESI -> IIP**	0.108	0.106	0.044	2.445	0.023	0.015	0.012	0.184	Supported
**SSI -> ESI**	0.036	0.040	0.073	0.491	0.001	0.623	−0.096	0.188	Not Supported
**SSI -> IIP**	0.573	0.571	0.051	11.262	0.540	0.000	0.474	0.666	Supported
**TI -> ESI**	0.570	0.570	0.066	8.590	0.271	0.000	0.441	0.697	Supported
**TI -> IIP**	0.244	0.248	0.064	3.826	0.077	0.000	0.131	0.373	Supported
**TI -> SSI**	0.679	0.683	0.035	19.380	0.855	0.000	0.611	0.744	Supported

Source: Authors’ own contribution based on SmartPLS.

## Data Availability

The data presented in this study are available on request from the corresponding author. The data are not publicly available due to Koudoua Ferhati that is a PhD Student and she we will use the data for her final thesis.

## Appendix A

**Table A1:** Social Sustainability Indicators (SSI).

Indicators	Definition
A1—Patient satisfaction	It is an important indicator of healthcare performance [51], and it measures the degrees to which the medical service responds to patients’ expectations [52].
A2—Average hospital stay	It shows how quickly medical staff can diagnose and prescribe treatment that does not require a further stay. Moreover, helps the facility predict how many patients they can bring into the facility during a specific period [53].
A3—Hospital readmission rate	This indicator calculates the rate of patients who come back to the facility shortly after they were seen. If high, it can indicate a lack of staff, experience, or attention during [53].
A4—Patient waiting time	It indicates the time a patient waits in a facility’s waiting room before being seen by one of the medical staff. It measures the average length of time patients spend in the hospital, per admission category [54].
A5—Patient safety	This indicator displays a healthcare facility’s ability to keep patients safe from contracting infections, complications, and other issues by tracking it in a recorded period that helps to recognize what areas are causing issues that can be improved [55].
A6—Waiting time in the emergency room	It gauges the time that the patient stays waiting in the emergency room until he gets a care service in the area [53].
A7—Number of new patients	This indicator measures the number of unique individuals who were first-time patients during the reporting period [53].
A8—Rate of vacant patients in beds	It shows the average rate at which beds in the facility are vacant [53].
A9—The patient transfer rate to other facilities	It tracks the number of patients being transferred to other healthcare facilities during the reporting period.
A10—Patient complaint rate	It logs the number of complaints filed by patients before, during, or after their period of care.

## Appendix B

**Table A2:** Economic Sustainability Indicators (ESI).

Indicators	Definition
B1—Average care costs	It is an image of the average out of pocket cost paid by the patient to the healthcare facility.
B2—Costs per payer	It reflects the averages of the cost that the patients pay for care services
B3—Average maintenance costs	Measures the direct and indirect costs to maintain equipment throughout the facility.
B4—Current cost per bed	Averages of the cost that the facility incurs for a patient’s entire stay.
B5—The cost of drugs and equipment	The cost that the facility pays for the total of drugs, medicines, and medical equipment during the reporting period.
B6—Average hospital expenses	Records the overhead expenses for direct operations of the facility. They affect the pricing of services.
B7—Cost of work-staff	Assembles the total cost of salaries, wages, and employee benefits. It affects the price of treatment for patients as well as the satisfaction of employees [53].
B8—Average care costs of insured patients	Averages of the cost that the facility incurs for a patient’s care after the elimination of costs % paid by insurant.

## Appendix C

**Table A3:** Indicators of Internal Process (IIP).

Indicators	Definition
C1—Medication errors	It can be used to improve the medical process by reporting mistakes made in the medication of inpatient and outpatient services [53].
C—2 Bed occupancy rate	Assesses the number of the occupied beds in the facility from all the departments divided by the number of actually available beds by a predefined number of days recorded [56].
C3—Mortality rate	A critical indicator that measures the number of deaths by the actual number of patients per the time of measuring the metric [57].
C4—Infection rate	It’s an indicator used in healthcare facilities to predict the probability of being infected, it measures the frequency of occurrence of new infection cases within patients during the recorded period [58].
C5—Clinical errors	Measures the frequency of clinical mistakes in the facility, which indicated the medical staff and equipment’s effectiveness [53].
C6—Medical errors	Measures the frequency of making a mistake of medications or dosage in prescribing medication within the facility [53].
C7—Waiting time for admission to the operating room	Measures. The mean time from presentation to the emergency department to the first surgical consultation [59].
C8—The average length of stay in the emergency room	Measures the average time from the patients arrive at the ER until discharge aftercare service in the facility [60].
C9—Laboratory test time	Measures the average amount of time it takes to run a test in the laboratory [53].

## Appendix D

**Table A4:** Technical Indicators (TI).

Indicators	Definition
D1—Quality of the building envelope	Measures the efficiency of the technical metrics with the purpose to improve the quality of *envelope* indicator in a recording period [61].
D2—Natural light penetration	Measures the amount and distribution of natural light needed for the good practices inside the healthcare facility’s different spaces [62].
D3—Degree of thermal comfort	Measures the degree of satisfaction with the thermal environment inside the facility [63] dissatisfaction may be caused by the thermal non-comfort of the body expressed by the medical staff or the patients, which could be tracked with the PMV and PPD indices [64].
D4—Hierarchy of functional spaces	Measures the degree of movement’s flexibility of the medical staff inside the facility, expresses the existence and the strength of direct and indirect needed relations between the spaces and departments.
D5—Distributions of medical devices	The metric assesses the availability and the distribution of the medical devices in the facility during the recorded period.
D6—Sufficient air conditioning	Measures the sufficiency of air out of an HVAC system compared to the standard norm for the room volume and the general well-being of staff and patients inside the facility.
D7—Vertical circulation	Assesses the availability and the distribution of automatic and non-automatic ways of vertical circulation inside the healthcare facility.
D8—Water consumption	Measures the average individual water usage calculated on a daily basis, at the facility’s level to provide an indication of the water consumption level and set the facility’s needs and strategies [65].
D9—Artificial lighting	Measures the sufficiency and the distribution of the artificial lighting depending on technical metrics: number of units, power per unit, and type of lamps used for each space to meet the minimum requirements [66].
D10—Energy and emissions control	Measuring the total energy consumption in the facility as well as the Greenhouse gas emission (GHGs) by setting tracking counters in the facility [67].
D11—Waste management	Measures the amount of waste collected and/or recycled by the facility, estimated intones. It takes into account any waste specific to the healthcare activities [68]. “Healthcare waste (HCW) is a by-product of healthcare that includes sharps, non-sharps, blood, body parts, chemicals, pharmaceuticals, medical devices and radioactive materials and toxic materials” [69] (p. 150).
D12—Location of the facility	Location is the physical geographical positioning of the facility; the indicator assesses metrics of the distance between the facility and main residential places, availability of public transportation to arrive at the facility, quality of the roads to be used by ambulances, and accessibility [70].
D13—Indoor air quality	Measuring this indicator allows maintaining the optimum indoor air quality using ventilation standards, Perceived Air Quality: (PIAQ) by having feedbacks from people’s perceptions of indoor air quality and particle measurements: PM.
D14—Acoustic insulation	Measuring the ability of sound insulation % for the use of the rooms and spaces that prevents unreasonable disturbances, for the Protection against disruptive noises has a significant impact on the well-being and the good practices environment for patients and staff.

## Appendix E

**Table A5:** Code, Factor Loading and Variance Inflation Factor of KPIs.

KPIs	Code	Factor Loading (FL)	Variance Inflation Factor (VIF)
**Social Sustainability Indicators**			
Patient satisfaction	A1	0.762	2.488
Average hospital stay	A2	0.713	2.353
Hospital readmission rate	A3	0.710	1.897
Patient waiting time	A4	0.773	2.291
Patient safety	A5	0.736	1.907
Waiting time in the emergency room	A6	0.731	2.152
Number of new patients	A7	0.705	2.291
Rate of vacant patients in beds	A8	0.719	2.229
Patient transfer rate to other facilities	A9	0.738	2.103
Patient complaint rate	A10	0.717	1.990
**Economic Sustainability Indicators**			
Average care costs	B1	0.771	2.651
Costs per payer	B2	0.719	2.289
Average maintenance costs	B3	0.736	2.356
Current cost per bed	B4	0.866	3.627
The cost of drugs and equipment	B5	0.756	2.063
Average hospital expenses	B6	0.846	2.747
Cost of work-staff	B7	0.749	2.391
Average care costs of insured patients	B8	0.851	2.808
**Indicators of Internal Process**			
Medication errors	C1	0.822	2.885
Bed occupancy rate	C2	0.727	1.900
Mortality rate	C3	0.789	3.389
Infection rate	C4	0.770	3.317
Clinical errors	C5	0.847	3.808
Medical errors	C6	0.860	3.608
Waiting time for admission to the operating room	C7	0.730	2.232
Average length of stay in the emergency room	C8	0.726	2.264
Laboratory test time	C9	0.720	1.921
**Technical Indicators**			
Quality of the building envelope	D1	0.715	2.611
Natural light penetration	D2	0.727	2.508
Degree of thermal comfort	D3	0.717	2.581
Hierarchy of functional spaces	D4	0.706	2.261
Distributions of medical devices	D5	0.721	2.207
Sufficient air conditioning	D6	0.749	2.565
Vertical circulation	D7	0.763	2.393
Water consumption	D8	0.708	2.328
Artificial lighting	D9	0.713	2.101
Energy and emissions control	D10	0.703	2.477
Waste management	D11	0.701	2.902
Location of the facility	D12	0.757	3.112
Indoor air quality	D13	0.798	3.852
Acoustic insulation	D14	0.706	2.593

## Appendix F

**Table A6:** The importance of KPIs in pandemic crisis.

KPIs	Code	Coefficient	Rank1	Rank2
**Social Sustainability Indicators**				
Patient satisfaction	A1	0.964	3	14
Average hospital stay	A2	0.980	1	4
Hospital readmission rate	A3	0.894	5	23
Patient waiting time	A4	0.977	2	7
Patient safety	A5	0.828	7	32
Waiting time in the emergency room	A6	0.894	5	23
Number of new patients	A7	0.728	10	38
Rate of vacant patients in beds	A8	0.815	8	33
Patient transfer rate to other facilities	A9	0.930	4	18
Patient complaint rate	A10	0.746	9	37
**Economic Sustainability Indicators**				
Average care costs	B1	0.841	6	30
Costs per payer	B2	0.695	8	40
Average maintenance costs	B3	0.793	7	34
Current cost per bed	B4	0.948	3	17
The cost of drugs and equipment	B5	0.885	5	27
Average hospital expenses	B6	0.901	4	22
Cost of work-staff	B7	0.959	2	15
Average care costs of insured patients	B8	0.966	1	12
**Indicators of Internal Process**				
Medication errors	C1	0.967	5	10
Bed occupancy rate	C2	0.785	9	35
Mortality rate	C3	0.922	6	19
Infection rate	C4	0.984	3	3
Clinical errors	C5	0.993	1	1
Medical errors	C6	0.913	7	20
Waiting time for admission to the operating room	C7	0.891	8	26
Average length of stay in the emergency room	C8	0.987	2	2
Laboratory test time	C9	0.980	4	4
**Technical Indicators**				
Quality of the building envelope	D1	0.893	8	25
Natural light penetration	D2	0.967	4	11
Degree of thermal comfort	D3	0.958	6	16
Hierarchy of functional spaces	D4	0.650	14	41
Distributions of medical devices	D5	0.711	13	39
Sufficient air conditioning	D6	0.969	2	8
Vertical circulation	D7	0.966	5	13
Water consumption	D8	0.878	10	29
Artificial lighting	D9	0.881	9	28
Energy and emissions control	D10	0.841	11	30
Waste management	D11	0.902	7	21
Location of the facility	D12	0.969	2	8
Indoor air quality	D13	0.980	1	4
Acoustic insulation	D14	0.754	12	36
